# Phenotypic spectrum of tinnitus patients bearing rare *ANK2* gene variants

**DOI:** 10.1007/s00405-024-08561-9

**Published:** 2024-03-20

**Authors:** Juan Martin-Lagos, Alberto Bernal-Robledano, Patricia Perez-Carpena, Mar Lamolda, Alba Escalera-Balsera, Lidia Frejo, Jose A. Lopez-Escamez

**Affiliations:** 1 Otology and Neurotology Group CTS495, Instituto de Investigación Biosanitaria, ibs.GRANADA, Universidad de Granada, 18071 Granada, Spain; 2 Division of Otolaryngology, Department of Surgery, Instituto de Investigación Biosanitaria, ibs.GRANADA, Universidad de Granada, Granada, Spain; 3 Department of Otorhinolaryngology, Hospital Clínico Universitario San Cecilio, Instituto de Investigación Biosanitaria, ibs.Granada, Granada, Spain; 4 Sensorineural Pathology Programme, Centro de Investigación Biomédica en Red en Enfermedades Raras, CIBERER, Madrid, Spain; 5 Department of Otolaryngology, Hospital Universitario Virgen de las Nieves, Instituto de Investigación Biosanitaria, ibs.Granada, Granada, Spain; 6 Meniere’s Disease Neuroscience Research Program, Faculty of Medicine and Health, School of Medical Sciences, The Kolling Institute, University of Sydney, Rm 611024, Level 11 Kolling Institute | 10 Westbourne St, St Leonards, Sydney, NSW 2064 Australia

**Keywords:** Tinnitus disorder, Phenotype, Exome sequencing, Genetics

## Abstract

**Purpose:**

To describe the clinical, audiological, and psychometric features observed in patients with chronic tinnitus and rare variants in the *ANK2* gene.

**Methods:**

We report a case series of 12 patients with chronic tinnitus and heterozygous variants in the *ANK2* gene. Tinnitus phenotyping included audiological (standard and high-frequency audiometry, Auditory Brainstem Responses (ABR) and Auditory Middle Latency Responses (AMLR)), psychoacoustic and psychometric assessment by a Visual Analog Scale (VAS) for tinnitus annoyance, the Tinnitus Handicap Inventory (THI), the test on Hypersensitivity to Sound (THS-GÜF), the Patient Health Questionnaire (PHQ-9), the Hospital Anxiety and Depression Scale (HADS) and the Montreal Cognitive Assessment (MoCA).

**Results:**

All patients reported a persistent, unilateral noise-type tinnitus, mainly described as white noise or narrowband noise. Seven patients (58%) were considered to have extreme phenotype (THI score > 76), and all patients reported some degree of hyperacusis (THS-GÜF score > 18 in 75% of patients). Seven patients scored MoCA < 26, regardless of the age reported, suggesting a mild cognitive disorder. ABR showed no significant differences in latencies and amplitudes between ears with or without tinnitus. Similarly, the latencies of Pa, Pb waves, and NaPa complex in the AMLR did not differ based on the presence of tinnitus. However, there were statistical differences in the amplitudes of Pa waves in AMLR, with significantly greater amplitudes observed in ears with tinnitus.

**Conclusion:**

Patients with *ANK2* variants and severe tinnitus exhibit an endophenotype featuring hyperacusis, persistent noise-like tinnitus, high-frequency hearing loss, and decreased amplitudes in AMLR. However, anxiety, depression, and cognitive symptoms vary among individuals.

## Introduction

Tinnitus, often described as a persistent and enigmatic auditory phenomenon, is a prevalent condition afflicting millions of people worldwide [1]. This condition is characterized by the perception of persistent ringing, buzzing, hissing, or other phantom sounds in the ears without any external source of such noise. Tinnitus can manifest as a debilitating disorder that significantly impairs a person’s quality of life, as the sounds are persistent and often difficult to ignore. Its complexity lies in its diverse causes and the subjective nature of the experience, making it a challenging condition to diagnose and treat effectively. Tinnitus is not merely an isolated auditory experience but a multifaceted disorder with physical, emotional, and cognitive consequences [2]. Many individuals with tinnitus report difficulties in attention and sleeping and experience heightened anxiety or stress due to the constant noise in their heads [3]. Its etiology can range from exposure to loud noise and ear-related conditions to underlying medical issues or even stress and anxiety, making it crucial for healthcare professionals to conduct thorough assessments to determine the root cause of a patient’s tinnitus.

Tinnitus is also considered the result of exacerbated plasticity of the central auditory system in response to the crosstalk with auditory nerve fibers [4]. The development of tinnitus is related to increased excitability in the auditory pathway, particularly the cochlear dorsal nuclei, but also the inferior colliculus, the medial geniculate body, and the auditory cortex. The perception of tinnitus involves different brain areas and neural networks in which other structures, such as the hippocampus or prefrontal cortex [5]. This complex combination has hampered advancements in the field, and the identification of a genetic contribution to tinnitus has been at the forefront of tinnitus research in the last few years.

Epidemiological studies involving twins and adoptees have provided substantial evidence supporting the hereditary component of tinnitus, particularly in cases of severe and bilateral tinnitus. Furthermore, familial aggregation of severe tinnitus has been documented, with a notable predisposition among women [6]. Nevertheless, our understanding of the molecular genetics underlying tinnitus remains in its early stages. While case–control studies have been conducted, many of these investigations were underpowered and needed more reproducibility [3].

Exome sequencing has been set to outline the tinnitus phenotype, focusing on individuals exhibiting extreme symptomatology. This approach has aimed to identify rare genetic variants within coding regions [7]. As a result of such efforts, the *ANK2* gene has been associated with severe tinnitus, shedding light on the involvement of membrane trafficking and cytoskeletal protein binding in the pathophysiology of this condition [8]. The *ANK2* gene encodes ankyrin-B, a cytoskeleton scaffolding protein that suppresses axon collateral branching and prevents microtubule invasion of nascent axon branches through direct microtubule interaction. Consequently, mutations in this gene may exacerbate axonal branching, leading to ectopic neuronal connectivity and an increased number of excitatory synapses. This could contribute to enhanced connectivity between auditory and non-auditory brain regions, particularly the para-hippocampus, implicated in tinnitus [9].

This study aims to report the clinical, audiological, and psychometric findings observed in patients with chronic tinnitus and rare variants in the *ANK2* gene to define an endophenotype in the carriers of rare variants.

## Methods

### Patient selection

We describe the audiological, psychoacoustic, and psychometric phenotype in a case series of adult patients with chronic tinnitus and rare variants in the *ANK2* gene that were selected from the exome sequencing Meniere disease and tinnitus databases generated by our group [8, 10]. We conducted the study following principles established in the Declaration of Helsinki, the UNESCO Universal Declaration on the human genome and human rights, and the requirements specified in the Spanish legislation in biomedical research, personal data protection, and bioethics. All participants received detailed information about the purpose of the study and expected outcome and signed a specific informed consent.

Participants were recruited at the Department of Otorhinolaryngology, Hospital Clínico Universitario San Cecilio, and Hospital Universitario Virgen de las Nieves. Blood or saliva samples were obtained to extract DNA to perform exome sequencing as described elsewhere [11]. Bioinformatic analyses were performed following the reported pipeline to generate FASTQ files for each participant; the paired-end sequences were mapped to the GRCh38/hg38 human reference genome, and single nucleotide variants were called into VCF files as previously reported [8].

The inclusion criteria were individuals > 18 of European origin diagnosed with tinnitus and rare missense variants in the *ANK2* gene. Patients with an associated otological disease or from a different population were excluded from the study.

### Psychoacoustic characterization

A comprehensive audiological study of all participating subjects was conducted within a sound-attenuated booth (Audiometric test booth S40-A, Sibelmed, Barcelona, Spain), including Pure-Tone audiometry (125–8000 Hz), Pure-Tone High-frequency audiometry (9000–20,000 Hz) and acufenometry. For this, a clinical audiometer AC40 (Interacoustics, Middelfart, Denmark) with A P4493 supra-aural headphones (RadioEar, Middelfart, Denmark) for conventional frequencies and a DD450 circumaural headphones (RadioEar, Middelfart, Denmark) for high frequencies were used. All audiological equipment was calibrated according to the manufacturer’s recommendations and the ISO 389-1 [12] and IEC 60645-1 standards [13]. Transducers were calibrated according to ISO 389-1. The auditory thresholds were determined according to the ascending method established in ISO 8253-1 (2020) [14].

Pitch and loudness were presented at different frequencies to assess tinnitus psychoacoustics on each individual. Participants must decide which stimulus is closer to their tinnitus pitch (noise). Subsequently, loudness is determined by comparing stimuli with different loudness levels (5 dB steps, starting from the hearing threshold). Later, the masking sound was presented at the frequency identified in the tinnitus pitch-matching procedure, starting from the hearing threshold. Patients were asked to report when the masking sound, presented in the examined ear, was loud enough to make their tinnitus not audible. The sound intensity was raised in an ascending way until an adequate level to cover the tinnitus was reached, thus defining the minimal masking level (MML). Finally, residual inhibition in the affected ear was examined. A positive inhibition was considered when tinnitus intensity decreased or completely disappeared for a minimum duration of 20 s.

### Psychometric characterization

A Visual Analogue Scale (VAS) [15] and two questionnaires were used to measure tinnitus’ impact on the patient’s quality of life and noise sensitivity, respectively: the Tinnitus Handicap Inventory (THI) [16] and the Hyperacusis Spanish version test (THS-GÜF) [17]. Patients with a THI score ≥ 76 were classified as extreme phenotype (EP); while individuals with a THI score ≥ 56 and < 76 were defined as almost extreme phenotype (AEP) [8]. In addition, two standardized depression and anxiety tools were used: the Patient Health Questionnaire (PHQ-9) [18] and the Hospital Anxiety and Depression Scale (HADS) [19]. Finally, the Montreal Cognitive Assessment (MoCA) [20] was used to evaluate a mild cognitive dysfunction.

### Auditory evoked short and middle latency responses

Electrophysiological recordings were performed using the Eclipse EP25 (Interacoustics^®^). Auditory Brainstem Responses (ABR) and Auditory Middle Latency Responses (AMLR) were obtained according to the parameters defined in the UNITI Protocol of study [21] and established by Manta et al. [22].

The ABR test measured the latencies of waves I, III, and V and the amplitudes of waves I and V evoked at an intensity of 70 dB. The components of the AMLR studied were the latencies and amplitudes of wave Pa, and the NaPa complex evoked at an intensity of 50 dB. These measurements were compared between ears with tinnitus and those without tinnitus. The type of stimulus used to record ABR waveforms was a click, and the repetition rate was 22 stimuli per second at an intensity level of 70 dB nHL. The recorded signal was filtered with a high-pass filter set at 33 Hz, 6 dB/octave, and a low-pass filter set at 1500 Hz, and the sample rate was 30 kHz. For recording AMLR waveforms, the stimulus used was one 2 kHz tone-burst with a duration of 28 sine waves, presented at a rate of 6.1 Hz/s and an intensity level of 50 dB nHL. The recorded signal was filtered with a high-pass filter set at 10 Hz, 12 dB/octave, and a low-pass filter set at 1500 Hz, and the sample rate was 3 kHz.

### Statistical analysis

Data were analyzed with IBM SPSS Statistics Base 28.0 software (Armonk, NY). The means of latency and amplitude values of ABR and AMLR in tinnitus-affected ears versus those without tinnitus were compared. The Mann–Whitney U test was used to compare variables between groups, with a value of* p* < 0.05 considered significant.

## Results

### Demographics

**Fig. 1:**
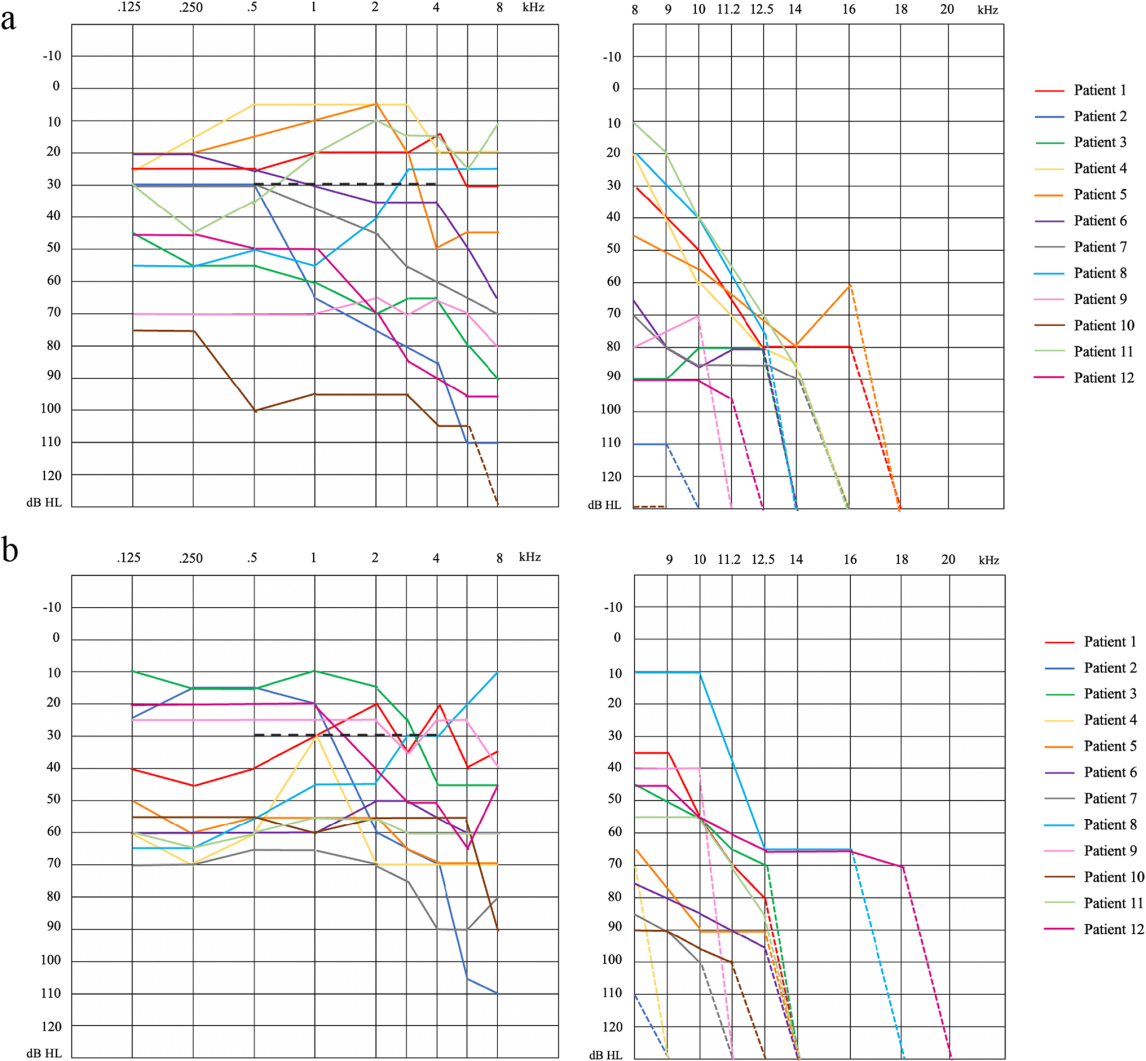
**a** Pure-tone audiometry (left) showing hearing thresholds (dB HL) for right ears in the frequency spectrum from 125 to 8000 Hz (0.125–8 kHz). High-frequency Pure-Tone audiometry (right) showing hearing thresholds (dB HL) for right ears in the frequency spectrum from 8000 to 20,000 Hz (8–20 kHz). **b** Pure-Tone audiometry (left) showing hearing thresholds (dB HL) for left ears in the frequency spectrum from 125 to 8000 Hz (0.125–8 kHz). High-frequency Pure-Tone audiometry (right) showing hearing thresholds (dB HL) for left ears in the frequency spectrum from 8000 to 20,000 Hz (8–20 kHz). The black dashed line shows the threshold for defining hearing loss

The case series consisted of 12 patients with tinnitus and heterozygous variants in the *ANK2* gene (Table 1), ages between 46 and 72 (57 ± 7.9), ten women, and two men. According to the criteria for hearing loss as defined in the UNITI Protocol of study [21], ten patients had bilateral hearing loss, and two had unilateral hearing loss, but all patients (*n* = 12) had high-frequency hearing loss (Fig. 1). Furthermore, all patients had lasting tinnitus (ten patients > 20 years and two subjects between 10 and 15 years).

**Table 1:** Variants carried by the studied individuals in the *ANK2* gene

ID	Variant	Protein	Consequence	CADD score	Allelic frequency			Patient
					gnomAD	gnomAD NFE	CSVS	
rs3112980	NC_000004.12:g.113145983G > A	–	Intronic	7.637	1.02E-01	1.48E-01	1.49E-01	1, 8, 10, 11
rs7689214	NC_000004.12:g.113145989G > A	–	Intronic	18.330	2.13E-01	1.87E-01	1.52E-01	9, 11
rs29341	NC_000004.12:g.113292475C > T	NP_001139.3:p.Val779 =	Synonymous	10.040	3.45E-02	3.87E-02	3.60E-02	4, 9
rs3736575	NC_000004.12:g.113336045C > T	NP_001139.3:p.Arg1193 =	Synonymous	0.513	2.34E-01	1.01E-01	1.03E-01	1, 3, 4, 8
rs149678604	NC_000004.12:g.113353074G > C	NP_001139.3:p.(Val1486Leu)	Missense	18.120	6.63E-04	4.96E-04	1.00E-03	2, 11
rs33966911	NC_000004.12:g.113354087C > T	NP_001139.3:p.Pro1823 =	Synonymous	1.474	7.55E-02	1.14E-01	1.15E-01	1, 5, 8
rs3796928	NC_000004.12:g.113354786C > G	NP_001139.3:p.Leu2056 =	Synonymous	0.462	1.10E-01	2.32E-02	3.60E-02	4
rs28377576	NC_000004.12:g.113355724 T > C	NP_001139.3:p.(Val2369Ala)	Missense	3.796	1.26E-01	1.18E-01	1.25E-01	1, 5, 8, 12
rs3733615	NC_000004.12:g.113355728A > G	NP_001139.3:p.Gln2370 =	Synonymous	5.746	2.76E-01	1.58E-01	1.83E-01	1, 3, 4, 5, 8
-	NC_000004.12:g.113355946 T > G	NP_001139.3:p.(Leu2443Arg)	Missense	28.100	–	–	–	1
rs3733617	NC_000004.12:g.113357121C > T	NP_001139.3:p.(Pro2835Ser)	Missense	0.634	1.43E-01	3.98E-02	5.80E-02	3, 4
rs10013743	NC_000004.12:g.113358266A > G	NP_001139.3:p.Glu3216 =	Synonymous	0.677	1.26E-01	1.18E-01	1.24E-01	1, 5, 8
rs34270799	NC_000004.12:g.113358518C > A	NP_001139.3:p.(Ser3300Arg)	Missense	24.000	1.96E-02	2.93E-02	2.50E-02	5, 9, 10
rs66785829	NC_000004.12:g.113365051 T > A	NP_001139.3:p.(Val3634Asp)	Missense	24.100	1.98E-03	2.57E-03	4.00E-03	12
rs2293324	NC_000004.12:g.113373152 T > C	NP_001139.3:p.His3891 =	Synonymous	0.014	2.99E-01	1.57E-01	1.85E-01	1, 3, 4, 5, 11
rs45454496	NC_000004.12:g.113373381G > A	NP_001139.3:p.(Glu3931Lys)	Missense	23.300	2.30E-03	3.44E-03	3.00E-03	7
rs35446871	NC_000004.12:g.113381478C > T	–	3’ UTR	5.291	3.04E-02	3.33E-02	4.10E-02	3, 6, 8

*ID* Reference Single Nucleotide Polymorphism identifier, *gnomAD* Global population from Genome Aggregation Database, *gnomAD NFE* Non-Finish European population from Genome Aggregation Database, *CSVS* Spanish population from Collaborative Spanish Variant Server, *UTR* untranslated region

### Psychoacoustic assessment

All individuals reported persistent, unilateral tinnitus and always ipsilateral with hearing loss (Table 2). During the Tinnitus Pitch / Loudness Match procedure, eight patients referred to their tinnitus as a white noise, and four were able to define it as narrowband sounds centered on various frequency spectrums (125, 250, 3000, and 6000 Hz, respectively). The loudness match presented a 2–16 dB SL variation with an average of 5.92 dB SL, while the MML varied between 3 and 25 dB SL with an average of 9.67 dB SL. Regarding residual inhibition, none of the patients showed complete inhibition of the tinnitus after exposure to noise for 1 min. Four patients reported a partial reduction in tinnitus, and the rest of the cases did not report any residual inhibition.

**Table 2:** Psychoacoustic assessment of patients with chronic tinnitus and missense variants in the *ANK2* gene

Patient	Tinnitus laterality	Tinnitus type and frequency (Hz)	Tinnitus threshold (dBHL)	Tinnitus loudness (dBSL)	MML (dBSL)	RI*
1	Left	WN	30	2	14	0
2	Right	NBN (125)	12	2	8	0
3	Right	WN	78	2	4	0
4	Left	NBN (6000)	58	4	8	0
5	Left	WN	66	5	8	0
6	Left	WN	52	16	25	0
7	Left	WN	75	3	9	1
8	Right	WN	26	10	14	0
9	Right	WN	69	1	3	1
10	Right	WN	92	10	8	0
11	Left	NBN (3000)	50	13	8	1
12	Right	NBN (250)	42	3	7	1

*WN* white noise, *NBN* narrow-band noise, *MML* minimum masking level, *RI (residual inhibition): 0 = negative; 1 = partial

### Psychometric evaluation

Regarding tinnitus distress, seven patients (58%) were considered to have extreme phenotype according to the THI score (Table 3). One patient reported a THI score of 56 to 76 (AEP), and the rest (*N* = 4) obtained scores < 56. According to the VAS test, ten patients scored ≥ 5, and five had values between 8 and 10, representing extreme discomfort with a high impact on the patient’s quality of life.

**Table 3:** Psychometric patients with chronic tinnitus and missense variants in the *ANK2* gene

Patient	VAS	THI	THS-GÜF	PHQ-9	HADS-A	HADS-D	HADS-T	MoCA
1	8	84	36	22	11	11	22	27
2	5	84	29	17	12	14	26	23
3	2	24	18	9	9	5	14	24
4	9	70	41	14	11	10	21	27
5	5	42	9	3	1	2	3	21
6	6	40	7	8	7	7	14	26
7	9	96	22	14	19	13	32	22
8	7	92	33	23	16	15	31	19
9	8	84	20	19	12	12	24	21
10	8	88	29	22	12	10	22	26
11	6	82	29	7	11	9	20	22
12	4	34	10	2	8	6	14	27
$\overline{x}$	6.42 $\pm$ 2.15	68.33 $\pm$ 25.72	23.58 $\pm$ 11.09	13.33 $\pm$ 7.46	10.75 $\pm$ 4.47	9.5 $\pm$ 3.90	20.25 $\pm$ 8.10	23.75 $\pm$ 2.80

*VAS* Visual Analogue Scale, *THI* Tinnitus Handicap Index, *THS-GÜF* Hyperacusis Spanish Version Test-Geräuschüberempfindlichkeit, *PHQ-9* Patient Health Questionnaire, *HADS* Hospital Anxiety and Depression Scale (*A* Anxiety, *D* Depression, *T* Total), *MoCA* Montreal Cognitive Assessment

All patients reported hyperacusis associated with their tinnitus. Nine out of 12 patients (75%) presented THS-GÜF scores > 18, which means that most of the subjects had a severe or very severe degree of disability attributed to hyperacusis.

Seven patients (58%) presented symptoms of moderate to severe depression, according to the PHQ-9 test; however, only two patients did not show symptoms of depression that potentially require therapeutic intervention (PHQ-9 < 4). According to the HADS anxiety subscale, 67% (*n* = 8) of the subjects studied presented clinically relevant anxiety symptoms (HADS-A score > 11). Only two patients did not present anxious characteristics. On the other hand, the depression subscale showed that 42% of patients (*n* = 5) had relevant symptoms (HADS-D score > 8) and, therefore, were patients with a probable diagnosis of depression. The rest of the patients did not show relevant depressive symptoms.

Finally, to estimate potential cognitive dysfunction, we used the MoCA test. The results showed that more than half of the patients (*n* = 7) scored < 26, indicating a probable mild cognitive disorder. Interestingly, contrary to what one might think, the mean age of these patients was lower (55.8 ± 9.7) than the mean age of patients with scores ≥ 26 (59.4 ± 4.6).

### Short and middle latencies auditory brainstem responses

In the ABR, latencies and amplitudes were not different between ears with or without tinnitus (Table 4, *p* > 0.05). Moreover, the latencies of the Pa, Pb waves, and NaPa complex in the AMLR did not differ according to the presence of tinnitus (*p* > 0.05). Conversely, although no differences were found in the amplitudes of the PaNa complex, the amplitudes of the Pa waves were significantly greater in ears with tinnitus.

**Table 4:** Electrophysiological Evaluation of patients with tinnitus and rare variants in the *ANK2* gene

	ABR			AMLR			
WAVE latency	Mean (SD) (ms)		*P* Between groups	WAVE latency	Mean (SD) (ms)		*P* Between groups
	Tinnitus *(N* = *12)*	No Tinnitus *(N* = *12)*			Tinnitus *(N* = *12)*	No Tinnitus *(N* = *12)*	
I	1.67 $\pm$ 0.31	1.54 $\pm$ 0.24	0.266	Pa	30.58 $\pm 4.$16	27.97 $\pm 3.$91	0.123
III	3.91 $\pm$ 0.32	3.69 $\pm$ 0.23	0.101	Pb	62.86 $\pm 7.$71	59.63 $\pm$ 7.93	0.346
V	5.71 $\pm$ 0.34	5.55 $\pm$ 0.27	0.178	NaPa	10.22 $\pm 4.$20	8.37 $\pm$ 2.58	0.314

WAVE amplitude	Mean (SD) (µV)		*P* Between groups	WAVE amplitude	Mean (SD) (µV)		*P* Between groups
	Tinnitus *(N* = *12)*	No Tinnitus *(N* = *12)*			Tinnitus *(N* = *12)*	No Tinnitus *(N* = *12)*	
I	0.09 $\pm$ 0.04	0.12 $\pm$ 0.08	0.378	Pa	0.61 $\pm$ 0.20	0.37 $\pm$ 0.23	0.021*
V	0.28 $\pm$ 0.15	0.37 $\pm$ 0.22	0.514	NaPa	0.83 $\pm$ 0.29	0.92 $\pm$ 0.40	0.674

Study of the latencies (ms) and amplitudes (µV) of the different components of the ABR (left) and AMLR (right) in ears affected by tinnitus and unaffected ears. *P value < 0.05

1 µV = 1000 nV

## Discussion

This study aims to describe the audiological and psychometric profile of patients with chronic tinnitus and rare variants in the *ANK2* gene. This study includes 12 patients reporting persistent severe tinnitus (THI > 56) since the onset of the disease; given that tinnitus may undergo variations over time, not all scores obtained during the duration of this study showed values > 56. However, our findings have demonstrated the persistence of high scores in the THI throughout the follow-up of most of these patients (83%).

Tinnitus is a common symptom in some diseases, but its closest relationship is undoubtedly established with hearing loss, including high-frequency sensorineural hearing loss, presbycusis, and Meniere’s disease (MD) [7]. In our series, 10 out of 12 patients were diagnosed with definite MD. Although more individuals with variants in *ANK2* need to be studied, we cannot rule out an effect of *ANK2* in hearing or vestibular loss in addition to tinnitus.

In addition to hearing impairment, other common psychological comorbidities such as depression, anxiety, insomnia, and cognitive impairment are present in 10–50% of tinnitus patients [23]. According to this, the standard assessment of these patients should include a complete audiological evaluation, psychoacoustic measures of tinnitus, and standardized questionnaires to determine the severity and its impact on health-related quality of life. Our study included the VAS scale as a reliable tool for measuring intensity, discomfort, and tinnitus-related distress [15]. In addition, the THI questionnaire was used to assess tinnitus severity and its functional impact on daily life [24]. THI and VAS scores show extreme annoyance values and a significant impact on the patient’s quality of life concerning tinnitus in more than half of the patients studied.

Depression and anxiety are commonly identified as contributing factors to the degree of distress in tinnitus sufferers [25]. Recently, De Ridder et al. proposed the concept of “tinnitus disorder” as distress associated with the conscious perception of noise, such as emotional distress, cognitive dysfunction, and autonomic excitation [2]. The literature has demonstrated the close relationship between depression and tinnitus [26, 27]. Bhatt et al. [28] reported a significantly higher prevalence of depression in patients who had suffered tinnitus in the last 12 months compared to those without tinnitus. They also demonstrate a higher prevalence of patients with both anxiety and tinnitus compared to the rates of patients with anxiety and no concomitant tinnitus, and they conclude that this association carries a strong relationship between tinnitus severity and the likelihood of anxiety and depression. Shargorodsky et al. [1] showed that about 50% of patients with anxiety disorders might suffer from tinnitus and that the prevalence of these disorders in tinnitus patients is higher compared to the general population.

To assess anxiety and depression, we have used the PHQ-9 scale, which is accepted as the best tool to identify the severity of symptoms in people with depressive disorder, and the HADS scale, which includes two subscales for anxiety and depression traits. Our results show that 58% of the patients presented symptoms of moderate to severe depression through the PHQ-9. However, only 42% of the patients presented relevant symptoms consistent with major depression in the HADS depression subscale, and 67% of patients showed anxiety symptoms based on the HADS anxiety subscale. As our sample includes patients with extreme phenotypes, these selection strategies are usually related to obtaining high scores in this type of psychometric test [7]. In addition, it is essential to note that the anxiety subscale of the HADS screens for anxious symptomatology with independence of its association with tinnitus. However, these questions can be easily related to feelings of tension, worry, or fear associated with tinnitus and not to a diagnosis of a generalized anxiety disorder.

Another relevant symptom observed in *ANK2* tinnitus patients was reduced tolerance to environmental sounds, also known as hyperacusis, in which the sounds are uncomfortably loud or painful, ultimately impairing social, occupational, and recreational activities [29]. Over 90% of people with hyperacusis report concurrent tinnitus, suggesting a strong relationship [30], and this relationship increases with tinnitus severity [31]. In our case series, all patients had hyperacusis related to their tinnitus, and 75% showed a high annoyance based on the THI score.

Cognitive impairment has been described in samples of tinnitus patients, and the severity of these symptoms has been correlated with the severity of tinnitus [32–34]. Wang et al. [35] demonstrated that the cognitive impairment occurring in patients with severe tinnitus was different from those occurring in patients with mild tinnitus, regardless of the degree of hearing loss, and suggested that the cognitive impairment may not secondary to the disease manifestations but a primary feature of the underlying disease. Melesci et al. [36] observed that in elderly patients with hearing loss, tinnitus appears to worsen cognitive dysfunction but concluded that it was still unclear whether cognitive impairment was a response to the manifestations of tinnitus or a feature of tinnitus in relation to age-related hearing loss. Our study used the MoCA test to detect mild cognitive dysfunctions. All of the patients from our series had bilateral hearing loss in different degrees, except for two patients with mild and moderate unilateral hearing loss. More than half of these patients had scores suggestive of mild cognitive impairment, with a lower mean age compared to the subgroup of patients without signs of cognitive impairment. However, we have not found statistically significant differences, probably due to insufficient sample size.

Average latency and amplitude data have been described for the different components of the ABRs [37]. However, depending on the hearing threshold, these values could vary in patients with hearing loss. It is now suggested that tinnitus arises as a dysfunction of central plasticity in response to decreased auditory sensory input after hearing damage. This leads to changes in the homeostatic control of gain in the auditory brainstem and auditory cortex and thalamocortical dysregulation [38]. Most of the patients in our series had bilateral hearing loss. In all cases, the ear affected by tinnitus also had different degrees of hearing loss. Edwall et al. [39] have described changes in the latencies and amplitudes of wave V in patients with chronic tinnitus as a result of the centrifugal effect of the auditory cortex [40] and subcortical brain structures beyond the brainstem. In our study, we observed a delay in the latencies of waves I, III, and V of the ears affected by severe tinnitus and a decrease in the amplitudes of waves I and V compared to the values obtained in the ears without tinnitus. Despite not showing statistical significance, these results align with the meta-analysis published by Milloy et al. [41], which described a generalized latency delay and amplitude decrease in the ABRs of tinnitus patients.

Although the role of cortical and subcortical auditory structures in the physiopathology of tinnitus needs to be better defined, only some studies have analyzed the role of AMLR latencies and amplitudes in tinnitus patients. Studying this type of mid-latency neural response can provide crucial information on the connection between the auditory pathways at the thalamocortical level and the mechanisms of corticolimbic dysfunction related to the degree of distress perceived by the patient with chronic severe tinnitus. Although the results are not statistically significant, our series shows delayed latencies in all AMLR components (Pa, Pb, and NaPb) of the ears with tinnitus. On the other hand, the amplitude of the main component of these auditory responses, Pa, is found in our series to be significantly increased in the tinnitus ears. It has been proposed that AMLRs may be a marker of tinnitus severity since both delayed latencies and increased amplitudes in diseased ears could reflect a malfunction of synchronized activity in subcortical and cortical regions [42].

### Limitations

The authors are aware of the limitations of this phenotyping study, including the low sample size, but participants were only selected if they had rare variants in the *ANK2* gene. This case series is focused on the analysis of patients with tinnitus extreme phenotype and rare variants in the *ANK2* gene, and these results cannot be extended to the general population. Furthermore, a control group to compare our results with a group of patients with defined MD will be needed to confirm the phenotype's audiological, psychoacoustic, and psychometric features.

## Conclusions

Patients with variants in *ANK2* and severe tinnitus have an endophenotype defined by hyperacusis, persistent noise type tinnitus, high frequency hearing loss, and increased AMLR amplitude. Anxiety, depression, and cognitive symptoms are also commonly observed, but they are not consistently found in all individuals.
